# A Review on Vehicle Classification and Potential Use of Smart Vehicle-Assisted Techniques

**DOI:** 10.3390/s20113274

**Published:** 2020-06-08

**Authors:** Hoofar Shokravi, Hooman Shokravi, Norhisham Bakhary, Mahshid Heidarrezaei, Seyed Saeid Rahimian Koloor, Michal Petrů

**Affiliations:** 1 Faculty of Engineering, School of Civil Engineering, Universiti Teknologi Malaysia, Skudai, Johor 81310, Malaysia; norhisham@utm.my; 2 Department of Civil Engineering, Islamic Azad University, Tabriz 5157944533, Iran; hooman.shokrav@gmail.com; 3 Institute of Noise and Vibration, Universiti Teknologi Malaysia, City Campus, Jalan Semarak, Kuala Lumpur 54100, Malaysia; 4 Faculty of Engineering, Universiti Teknologi Malaysia, UTM Skudai, Johor Bahru, Johor 81310, Malaysia; mheydarrezaei@gmail.com; 5 Institute for Nanomaterials, Advanced Technologies and Innovation (CXI), Technical University of Liberec (TUL), Studentska 2, 461 17 Liberec, Czech Republic; s.s.r.koloor@gmail.com (S.S.R.K.); michal.petru@tul.cz (M.P.)

**Keywords:** vehicle classification, vehicular ad hoc networks, weight-in-motion, global positioning system, light detection and ranging, ultrasonic, radar, video images

## Abstract

Vehicle classification (VC) is an underlying approach in an intelligent transportation system and is widely used in various applications like the monitoring of traffic flow, automated parking systems, and security enforcement. The existing VC methods generally have a local nature and can classify the vehicles if the target vehicle passes through fixed sensors, passes through the short-range coverage monitoring area, or a hybrid of these methods. Using global positioning system (GPS) can provide reliable global information regarding kinematic characteristics; however, the methods lack information about the physical parameter of vehicles. Furthermore, in the available studies, smartphone or portable GPS apparatuses are used as the source of the extraction vehicle’s kinematic characteristics, which are not dependable for the tracking and classification of vehicles in real time. To deal with the limitation of the available VC methods, potential global methods to identify physical and kinematic characteristics in real time states are investigated. Vehicular Ad Hoc Networks (VANETs) are networks of intelligent interconnected vehicles that can provide traffic parameters such as type, velocity, direction, and position of each vehicle in a real time manner. In this study, VANETs are introduced for VC and their capabilities, which can be used for the above purpose, are presented from the available literature. To the best of the authors’ knowledge, this is the first study that introduces VANETs for VC purposes. Finally, a comparison is conducted that shows that VANETs outperform the conventional techniques.

## 1. Introduction

The methods used in traffic engineering to derive vehicle parameters in their moving status are defined under the term of vehicle classification (VC). VC is a module used to categorize vehicles into several distinct classes. There are different definitions available for VC in the literature. Table 1 presents a summary of the existing definitions to further clarify the phenomenon. VC is a fundamental part of intelligent transportation systems and is widely used in various applications like the monitoring of traffic flow [1,2], automated parking systems [3,4], security enforcement [5], and even structural health monitoring [6,7,8,9,10]. In these methods, vehicles can be detected by passing through fixed sensors [11,12], passing through monitoring areas [13,14], global coverage [15,16], or a hybrid of these methods [12,17]. A wide variety of information can be extracted using sensors and detectors which may include vehicle count, shape—i.e., height, width and length—[14,18], speed [19,20], axle weight and spacing [21,22], acceleration/deceleration [23], make and model [24,25,26] and number plate [27,28].

The manual count is the simplest VC method. However, these methods are time-consuming and subject to errors. Vision-based methods are the most widely studied and used approaches for VC and traffic monitoring. Vision-based methods extract visual attributes such as the color, lines, and textural patterns as features in the video to detect and track vehicles [37]. Vision-based VC includes several steps such as image segmentation, feature extraction, training, and pattern recognition. The main purpose of image segmentation is to extract the object of interest from the background based on some useful cues such as pixel color [38,39], edges—obtained from image gradients [40]—and pixel intensity (gray level) [41,42]. Training data are used for the pattern recognition and classification stages. Particular care must be taken when using the vision-based methods to respect the privacy and anonymity of individuals involved [23]. A comprehensive review of the vision-based methods was conducted by Wang et al. [43].

Pneumatic tube detectors for VC were used first in 1920 and are being used today for the short-term collection of vehicular data [44]. A pneumatic tube can detect the number of axles and the axle spacing in a moving vehicle. This method is not suitable for high volume, high-speed roadways. A magnetic loop detector is a technology that has been used for VC in recent decades. The magnetic loop can be used for VC by detecting vehicle length [45,46]. Vehicle speed can be directly measured using dual-loop detectors [47,48]. Thought loop detectors are relatively inexpensive and perform automatic classification, but they do not do well in high congestion [23]. Piezoelectric sensors are used to detect the weight of the vehicle and the axle configuration [19,49]. The piezoelectric detectors are used alone or in combination with Weight-In-Motion (WIM) systems. The drawback of piezoelectric sensors, however, is their sensitivity to vehicle speed and pavement temperature. Radar sensors are a popular tool to extract classification through vehicle dimensions (length, size, height, etc.) [50,51]. Though radar sensors are less sensitive to environmental variation compared to other methods, they are not suitable for dense traffic congestions [23]. Infrared sensors measure the reflected infrared light by each vehicle and compare the data with the database to find the best-matched profile [52,53,54]. Infrared sensors are sensitive to environmental factors. Acoustic sensors use speed-independent acoustic signatures to identify vehicle classes [55].

WIM is an important source in traffic data collection and the classification of vehicles. The WIM architecture consists of two parts: modeling and estimation. The WIM system [56] was developed for measuring vehicle weight data. WIM is a system equipped with various sensors, digital cameras, and computers that is installed on a bridge structure. WIM measures the dynamic axle load of moving vehicles to obtain vehicle weight data. Multiple detection techniques are used in WIM for the accurate classification of vehicles [57,58]. However, WIM is a fixed-location weight measurement device and measures the axle weight just when its wheels pass over the sensors. On the other hand, the WIM system is expensive and not feasible for local roads.

It is shown that the methods based on the fixed location sensors could provide valuable information in combination with other methods [59,60]. The vision-based methods can provide information about the make and brand of a vehicle that could be used to extract other information such as gross weight and axle properties [61,62]. Besides the mobility state of vehicles, speed acceleration and direction also could be retrieved within the coverage area of the camera [59,63]. The studies state that the use of a GPS-based positioning system is the most reliable way to extract the locus and movement information; however, the methods lack information about the physical parameters of vehicles. Furthermore, in the available studies, smartphone or portable GPS apparatuses are used as the source of the extraction vehicle’s kinematic characteristics, which are not dependable for the tracking and classification of vehicles in real time. Overall, it can be concluded that the available methods are not a reliable choice for real time and global VC. Therefore, research into a new trend for cloud-based or vehicular networks as an alternative to traditional VC applications is an open field of study.

In recent decades, autonomous driving has drawn huge attention from both academia and industry and great effort has been put into designing vehicles with the capability to self-navigate urban streets [64,65,66,67,68]. Nowadays, autonomous and autopilot vehicles are fleeting on the roads and they are expected to revolutionize the transportation system in an unprecedented manner. The Google driverless car was the first autonomous case to be driven in an urban context [69]. The Daimler Smart EQ concept is another example of a fully automated vehicle in which a driver is no longer needed [70]. Tesla Motors developed a semi-autonomous vehicle, called Tesla autopilot, using artificial intelligence and hardware technology with real time driving updates [71]. Such vehicles are equipped with sensors, radars, cameras, and satellite feeds to collect, store, and analyze a tremendous amount of data relating to road traffic [69]. In this paper, the term “smart vehicle “ is used to denote a generic describer for a vehicle from which ambient data are automatically collected using sensing devices, then stored within a centralized onboard hardware unit for further processing. All autonomous or autopilot vehicles fall within this grouping.

Smart vehicles are an emerging application of automotive technology, capable of sensing and monitoring their surroundings and mobilizing on-demand services. Based on the USA National Highway Traffic Safety Administration (NHTSA), smart vehicles can be classified into five stages of autonomy that ranges from no automation (Level 0) to full automation (Level 5) [72]. The five stages of autonomy in smart vehicles, indicating the vehicle state and the role of the driver, and providing examples for each stage, are presented in Table 2. Smart vehicles are equipped with various sensors such as Light Detection and Ranging (LiDAR), radar, cameras, thermal imaging cameras, ultrasonic sensors, GPS receivers and Inertial Measurement Units (IMUs), which generate copious amounts of data every second [73,74].

The availability of the smart foundation and common tools for data collection and transmission, as well as access to an anonymous a privacy-preserving scheme for sharing information and exchanging data, have provided a unique combination of properties, making smart vehicles an attractive choice for many high-tech applications. VC can benefit from these technologies to a significantly greater extent.

Arguably, the adoption of smart technologies in the design and manufacturing of new vehicles motivated the development and adoption of intelligent systems for vehicle identification and classification. The existing VC approaches, except for GPS-based methods, generally have a local nature and can classify the vehicles if the target vehicle passes through fixed sensors, passes through the short-range coverage monitoring area, or a hybrid of these methods. Collecting real time traffic information as well as providing global access to sensor data are two crucial requirements for a reliable VC method.

The parameters of interest in VC methods generally count, shape—i.e., height, width and length—speed, axle weight and spacing, acceleration/deceleration. The present paper intended to study the available literature on VC methods to provide an overview of the topic. A study was conducted to investigate the potential methods that could detect, identify, and classify vehicles in a global and real time manner. The selected methods were shortlisted and the most suitable one was chosen for the feasibility study. The idea of “smart vehicles” refers to transportation means that are partially or fully driven by computers, taking advantage of various sensor platforms and cameras for collecting, storing, and sharing a huge amount of data.

Vehicular Ad Hoc Networks (VANETs) are an emerging part of the intelligent transport system that have aroused great interest worldwide in the last decade [75]. VANETs are a network of intelligent interconnected vehicles and are composed of an Onboard Unit (OBU) and a stationary access point, termed roadside units (RSUs) [76]. OBU is a device fitted to each vehicle, which basically includes memory, a processing unit, a GPS receiver, and an antenna for short-range Internet connection [77]. The OBU provides a vehicle to vehicle (V2V) communication or vehicles to RSU infrastructures (V2I) [78]. All transactional data during the trip are recorded within a hardware module called an event data recorder (EDR), which is a form of black box within the vehicle [79]. Each vehicle sends periodic data into its adjacent vehicles [80]. Privacy preservation and security assurance of the transmitted data are the most significant concerns in the appropriate application of network systems [81]. As a result, a secure anonymous key distribution mechanism is used among the trusted entities of the network. Each disseminated message contains content, a signature, and a certificate. The certificate of a received message is validated and the public key is used to decrypt the certificate and identity of the sender [82].

Different definitions are used to describe the VC phenomenon (See Table 1). While some researchers believe that VC methods presuppose the monitoring/tallying system and cannot receive exact information directly from each vehicle [35], others accept all methods that can classify vehicles into their respective types under the VC term [29,30]. The researchers in the first group who consider that VC cannot receive exact vehicular data believe that communication systems such as VANETs should not be considered as a particular technique for VC, while others have a less rigid definition of the phenomenon and consider using mobile networks such as VANETs as a particular class of VC methods. To respect both of these criteria simultaneously, the methods that use exact vehicular information are also included for potential future applications.

VANETs were capable of providing global information on vehicles in a real time manner. The provided information could be mobility parameters as well as physical vehicular parameters. The results of the feasibility study show that, in a VANET system, the mobility information—e.g., position, traveling lane, speed, and acceleration and deceleration—as well as the physical characteristic parameters of vehicles—e.g., weight, height and length—are used for a wide variety of applications such as parking management, traffic control, safety, and accident avoidance. Table 3 shows a summary of the literature reviews on VC.

The presented review of the available literature on VC shows that most of the research to date mainly focuses on vision-based methods. In these review papers, no particular attempts were performed to review the potential impact of smart technologies in vehicular networks and communication to enhance the efficiency and efficacy of VC systems. Nonetheless, there is only one review paper discussing other features than those used to capture the scene regularities and its focus is mainly on conventional VC methods. Some review papers have discussed vehicle-assisted VC but they only mentioned instruments like mobile sensory devices, such as GPS receivers and smartphones, in vague terms. Plus, these reviews discussed the limited aspects of each technique and the state-of-the-art vehicular sensing and communication technologies were neglected. A paper by Jain et al. [85] studied various traffic monitoring schemes. The vulnerabilities of these techniques were evaluated and the potential misuse of the information was discussed. In another research by Borkar and Malik [97] reviewed the application of acoustic signals to estimate vehicular speed, density, and classification. The study only focused on smart methods by utilizing efficient smartphones, cameras, drones, and robotic sensors. In other studies, such as those by Shukla and Saini [83], Yousaf et al. [84], and Daigavane et al. [86], vision-based techniques were the foci of the study.

For a period of nearly two years, a number of university academics and industry partners have been working on a project to develop “autonomous electric vehicles for transportation of goods and freight”. During the course of the work, the authors encountered various challenges pertaining to finding a reliable method to extract the mobility information parameters—e.g., position, traveling lane, speed, and acceleration/deceleration—as well as physical characteristics—e.g., weight, height and length—of fleeting vehicles in real time and in a global manner, as addressed in this manuscript. However, as the study was expanded, it was realized that there is not any review study that provides the same breadth and depth of knowledge we have come to expect to address the aforementioned objectives. As a result, a stepwise procedure was followed to evaluate the effectiveness of the existing methods used in the characterization, identification, and classification of vehicles in their normal operating conditions, on the one hand, and to investigate the new potential options that might provide solutions for on-road vehicle classification, on the other hand. The findings indicated that the available vehicle classification methods are unable to provide real time global physical and mobility data for on-road vehicles in their normal operating condition. The present review covers the literature on the topic and sheds light on potential innovative ideas to streamline and improve the quality and reliability of the extracted vehicular data. As far as the authors are aware, this is the first study that has provided this knowledge in the way we have presented it. The authors strongly believe that vehicle classification of on-road systems in a real time state using cloud-based or vehicular networks will be a new direction and open field of study for future research as an alternative to the current traditional vehicle classification approaches.

In the following sections, conventional VC methods are discussed. Afterward, the feasibility of using VANETs for VC application is investigated. In the next step, a roadmap is being drawn up in this paper, aimed at outlining how this vision could be developed as future work, and finally, a conclusion is given in Section 5.

## 2. Conventional Vehicle Classification Methods

**Figure 1:**
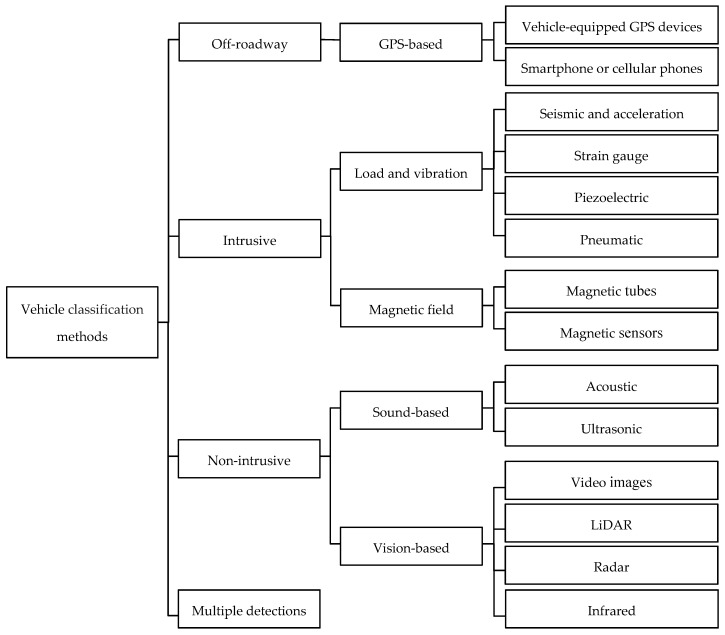
The methods used for VC.

VC methods can be divided based on the processing medium into three main groups: intrusive, non-intrusive, and off-roadway. Some of the available studies use a combination of the aforementioned methods, which is classified under the multi-detection category. The groups and subgroups of each class are presented in Figure 1.

Non-intrusive sensors are typically positioned next to or above the road of interest and, in some cases, a single sensor can be used for multiple lanes. The installation and maintenance of non-intrusive sensors are easier than intrusive sensors and the monitoring data are not affected by pavement quality [98]. Intrusive sensors are typically installed in holes on the road surface, by tunneling under the road surfaces or anchoring to the surface of the road [99]. Both intrusive and non-intrusive sensors are sensitive to adverse environmental conditions, the implementation is of high capital cost and they require expensive maintenance [100]. Off-roadway sensors are mobile sensors that can be employed via aircraft or satellite, or in vehicles equipped with GPS receivers [101]. Further details on the classification of sensors will follow.

### 2.1. Vision-Based Methods

Vision-based methods are widely studied for VC and the largest number of studies on fixed location VC belong to video image detection. The cameras used for collecting data can be surveillance video systems, omnidirectional cameras [102] aerial images [103,104], Closed-Circuit Television (CCTV) [105,106], or normal cameras [107,108]. These methods generally use image processing techniques for the detection, tracking, and classification of vehicles. The processing and classification of vehicles using video image detection includes several steps that generally include preprocessing, feature extraction and selection, and classification. Preprocessing is a step to enhance the quality of images. Image segmentation, shadow removal, and occlusion handling are of the most adapted methods for video image detection.

Image segmentation is one of the fundamental techniques in image processing. Velazquez-Pupo et al. [14] presented a high-performance vision-based system with a single static camera. In this approach, moving objects are first segmented by the Gaussian Mixture Model (GMM) and, after feature extraction, tracking is performed with a Kalman filter. The proposed system can be run in real time with an F-measure of up to 98.190%, and an F-measure of up to 99.051% for midsize vehicles. Chen et al. [109] used a recursively updated GMM for segmentation. A multi-dimensional smoothing transform is used to improve the segmentation performance. A kernelled support vector machine (SVM) is used to classify the model. Singh et al. [110] introduced a web-based online traffic management system using segmentation, blob analysis, and Motion History Image (MHI) methods in the processing stage. The proposed system traces the estimated density of vehicles in different locations at different times to assist in choosing a suitable path. Abinaya et al. [111] proposed a method to enhance the performance of video-based VC using a single standard camera. A robust video-based system to detect, track, classify and count vehicles using marker-controlled watershed segmentation, a Gabor filter and a support vector machine (SVM). The experimental results showed a significantly improved performance in the watershed segmentation in relation to vehicle detection. Audebert et al. [103] presented a deep learning-based segment-before-detect method to process the big data of a VC obtained from remote sensing. A deep, fully convolutional network was trained and the learned semantic maps were used for segmentation. A Convolutional Neural Network (CNN) was trained for VC. Zhang et al. [112] described an image enhancement process using threshold segmentation and noise elimination. Features are extracted using Gabor extraction, then a SVM is used for classification.

Shadow removal is an image processing step aimed at enhancing the quality of a video or image for computer systems. Jehad et al. [113] developed a fast vehicle detection and counting method using a video camera. A system is presented for extracting traffic data using video image processing using background subtraction, shadow removal, and pixel analysis. The results show that the algorithm is capable of counting 95% of the vehicles, even in the case of some shaking in the video feed. Asaidi et al. [114] presented two approaches to enhance automatic traffic surveillance systems. A contrast model is proposed to remove dynamic shadows. It is shown that the proposed approach outperforms other methods with a classification accuracy of 96.96% and a shadow elimination rate of 95–99%. Yang et al. [115] proposed a system to estimate traffic flow for different outdoor illuminations and cast shadows. A traffic monitoring system is proposed to improve image quality using foreground extraction, shadow discrimination and color and edge invariants. Yu et al. [116] proposed a length-based method for the real time classification of moving vehicles in multi-lane traffic video sequences. Background subtraction, edge-based shadow removal, thresholding segmentation algorithms are followed with the horizontal projection to classify vehicles. The experimental results show that the classification accuracies for the large and small vehicles are 97.1% and 96.7%, respectively. Meher et al. [117] proposed a method to enhance the quality of the vision-based VC by the detection and removal of moving shadows. The potentiality and superiority of the method were compared with the existing methods.

Occlusion handling is an image processing step for tracking the vehicle when it is in a partially occluded position. Moutakki et al. [118] presented an approach using occlusion handling tracking, and One-Class SVM (OC-SVM) classification. Velazquez-Pupo et al. [14] used a real time video surveillance system for the classification and counting of vehicles using the codebook model and occlusion handling. The histograms of oriented gradient followed by a SVM are used to classify vehicles by their type.

In the feature extraction step, suitable features for the classification of vehicles are selected. Texture features and shape features, including Scale-Invariant Feature Transform (SIFT) [119,120], Oriented Fast and Rotated Brief (ORB) [121,122], Speeded-Up Robust Features (SURF) [123,124], and Vehicle Make and Model Recognition (VMMR) [25,125], are among the most common features used for VC. Texture features are used to overcome the disadvantages of color and intensity features. Jayadurga et al. [126] enhanced the performance of vehicle classifiers in a highly textured background. A hybrid texture feature extraction, including statistical and spectral texture features, is used without pre-processing for classification. A classification accuracy of 90.1% was achieved and the result was compared with different methods from similar works in the literature. Chen et al. [105] applied a recursively updated GMM algorithm to identify vehicles based on their type and color using texture features. Multi-dimensional smoothing transform is used to improve the segmentation performance. Good recognition rates were achieved for pragmatic VC. The non-invasive features of SIFT are usually used to detect key points. The used characteristics in SIFT are invariant to lighting, enlargement, translation, and the rotation of images. Khanaa et al. [127] proposed SIFT and the Random Sample Consensus Algorithm (RANSAC) to classify road vehicles and upgrade characterization and counting. Ambardekar et al. [128] implemented a constellation approach using a dense representation of SIFT features for reliable VC of high inter-class variation using video surveillance. Three classes were considered: sedans, vans, and taxis. ORB is a descriptor that is faster than SURF and SIFT and less affected by image noise. Song et al. [129] proposed a trajectory clustering framework for vehicle analysis using the ORB algorithm. A matching method based on Hamming distance is used. Finally, a clustering method is proposed to classify vehicles. The accuracy of the proposed method can reach up to 95%. Furthermore, vehicle type can be estimated to realize VC. VMMR is a sophisticated vision-based application based on license plate recognition. Biglari et al. [28] proposed a cascaded part-based model for VMMR. This system uses a linear support vector machine (LSVM) for feature extraction. A cascading scheme is used to speed up the processing based on confidence and frequency. The proposed approach achieved an average accuracy of 97.01% on a challenging data set and an average accuracy of 95.55% on the CompCars data set. Siddiqui et al. [12] proposed and evaluated unexplored approaches for real time automated VMMR. Vehicles’ front- or rear-facing images are embedded into Based on SURF (BOSURF) histograms, which are used to train multiclass SVMs for classification. The experimental results prove the superiority of the proposed work in terms of both processing speed and accuracy.

VC is the final step for the identification of vehicle classes. Support vector machine (SVM) and Neural Network (NN) methods are widely used for the classification of the extracted features. In Table 4, some of the most common soft-computing methods used for pattern recognition, classification, training, or prediction in VC are presented.

There are some other classifiers and learning machines that are also used for VC, such as forest tree [145] nearest neighbor [146,147,148], decision tree learning [149], extreme learning machine classifier [55], genetic fuzzy classifier, [150] kernel principal component classifier [151] and histogram-based nonlinear kernel classifier [38]. For the cases where multiple sensors are used for the detection and classification of vehicles, the data are fused. Bayesian networks [105,152,153] are a common method for the fusion of the input data.

### 2.2. Remote Sensing Methods

Remote sensing methods are of the fastest growing trends in VC due to the global nature of the presented information by these methods. Radar is widely used for detecting moving objects on the ground, such as in traffic monitoring and VC. Aziz et al. [154] practiced passive forward scattering radar systems for VC. A Doppler signature is captured once the vehicle passes through the scattering region. The vehicles are separated based on size categories. Lee et al. [155] proposed a Frequency-Modulated Continuous Wave (FMCW) radar system to extract three distinctive signal features from vehicles’ cross-sections. SVM was used for the classification of the extracted features. Through the field measurement results, an accuracy higher than 90% was achieved. Abdullah et al. [50] examined Automatic Target Classification (ATC) for feature extraction. The combination of Z-score and NN is adapted for the classification of the extracted features. The obtained results demonstrate that an enhanced performance was achieved by using a large number of features. Chen et al. [156] employed Synthetic Aperture Radar (SAR) for the tracking and classification of vehicles. Target echo signals are decomposed into many Intrinsic Mode Functions (IMF) using ensemble empirical mode decomposition (EEMD). The experimental shows up to a 90% success rate for classification. Saville et al. [146] surveyed wide-band, wide-aperture, and polarimetric radar data for VC. A 10-VC experiment in the spectrum parted linked image test algorithm was used for the verification.

LiDAR is a remote sensing technology that can generate Doppler for detecting distributed or hard targets. LiDAR transmits and receives electromagnetic radiation and the extracted features from vehicles are analyzed after the processing of the data [157,158].

Thermal images display the amount of infrared energy emitted, transmitted, and reflected by a vehicle. Most of the thermal images are used for the detection of vehicles in the battlefield. Yang et al. [52] proposed a novel feature extraction method based on the Target Trait Context (TTC) to enhance the shortcomings of thermal images for VC. The validation results show that the proposed TTC feature outperforms the previous methods. Khamayseh et al. [53] proposed a robust framework for person–vehicle classification from infrared images. The traffic observations from an infrared smart surveillance system are collected by Situational Awareness (SA). The experimental results prove the effectiveness of the proposed framework. Mei et al. [54] introduced a visual tracking method by casting tracking as a sparse approximation problem. The approach was validated through a vehicle tracking and classification task using outdoor infrared video sequences.

Aerial images are a popular source of information in the remote sensing field. Aerial images have a high resolution and can cover a large area of interest. Several studies have focused on using aerial images for VC. Li et al. [104] employed Regions with a Convolutional Neural Network (R-CNN) features to recognize small vehicles from aerial images. Feature map selection and bi-partite main-side network construction were used to improve the performance. The effectiveness of the proposed network extension was verified by comparing it with its strong and similarly shaped counter-parts. Audebert et al. [103] presented a deep learning-based segment-before-detect method for segmentation, detection, and classification of vehicles in aerial images. A deep, fully convolutional network was trained and the learned semantic maps are used for segmentation.

### 2.3. Magnetic Sensors

Magnetic sensors can detect the distortion in the Earth’s magnetic field caused by a passing vehicle [159]. Magnetic loop detectors are the most commonly used sensors in VC and traffic monitoring [160]. Magnetic loops are generally installed in the form of single-loop detectors, dual-loop detectors, and asymmetrical shapes—e.g., rectangular loops. Several studies have researched the use of single-loop detectors for VC.

Lamas-Seco et al. [20] modeled an inductive loop detector to study the influence of significant vehicle characteristics on inductive signatures. The obtained results for both prototypes and the inductive sensor simulator exhibited similar characteristics, validating the model used in their work. Coifman et al. [161] refined non-conventional techniques for estimating speed with single-loop detectors. The obtained results from this method were compared with the ones obtained from video and dual-loop detectors. This work successfully leverages the existing investment deployed in single-loop detector count stations. Meta et al. [46] presented a VC method that uses the signal generated by a single inductive loop detector. A VC algorithm is introduced that take advantage of Discrete Fourier Transform (DFT), Principal Component Analysis (PCA) and backpropagation neural network (BPNN) classifiers. The recognition rate was 94.21% for the VC.

Dual-loop detectors are formed by two consecutive single-loop detectors spaced several meters apart. These detectors are widely employed to obtain average speed, occupancy, and flow information in traffic management systems. Wu et al. [47] presented a method that considers the change in acceleration in a dual-loop detector. A new parameter was defined for unobserved acceleration. The method proposed to reduce the effect of acceleration change in dual-loop detectors. Analytically, it was shown that errors due to acceleration will not lead to errors in length class. The proposed approach reduced the classification error rate due to acceleration by at least a factor of four relative to the best conventional method. Wei et al. [162] presented a hybrid method to identify traffic phases using the variables obtained from dual-loop inductive sensors. The hybrid method incorporates the level of service approaches and K-means clustering methods to improve the clarification of the traffic flow phase. The result indicates that, compared with the existing models, the accuracy is increased from 42% to 92%. Li et al. [163] investigated statistical inference in relation to vehicle speed and vehicle length using dual-loop detector data. Statistical inference for vehicle speed and length was investigated by Bayesian analysis to set formulas for the online estimation of speed and length. The method was presented using real traffic data.

Inductive loops can also be asymmetrically shaped. Mocholí-Salcedo et al. [45] made a detailed study of the magnetic field generated by rectangular loops in traffic control systems. The inductance of numerically calculated magnetic loops is and the results are compared with the most commonly used empirical methods for inductance calculations. A great similarity between the empirical and numerical results was achieved. The magnetic signature of the inductive loop is widely used as a feature for the detection and classification of vehicles in inductive loops [45,160,164].

Magnetic sensors are less expensive and complex compared to magnetic loops and are highly favorable for VC. Several studies have studied magnetic sensors for VC in their works. Haj Mosa et al. [131] presented a truck detection algorithm involving one single sensor. A novel Soft Radial Basis Cellular Neural Network (SRB-CNN)-based concept is developed, validated, and benchmarked with a selection of the best representatives of the current related classification concepts. The proposed method fulfills the requirements regarding robustness, low-cost, high processing speed, low memory consumption, and capability. He et al. [165] proposed an approach to overcome the shortcomings of the conventional data aggregation from single-point sensor data. A filter–filter–wrapper model is adopted to evaluate and determine non-redundant feature subsets. C-support vector machines (C-SVMs) were established in parallel with particle swarm optimization (PSO) for VC. The results showed that the classification accuracy was over 99%. Šarčević et al. [166] presented an analysis of magnetic sensors implementable in a microcontroller system. A new classification method for a single magnetic sensor-based technique using the NN classifier is designed. Li et al. [142] proposed an online VC method using a magnetic sensor. Eight features are extracted, then the decision tree model is trained based on the Classification and Regression Tree (CART) algorithm with a Minimum Number of Split (MNS) samples. Finally, the trained decision tree model is pruned with a Minimum Error Pruning (MEP) rule. The results show that the proposed method enables online vehicle type classification with the advantages of high classification accuracy, sample robustness and less execution time. Yang and Lei [167] developed a vehicle detection system using low-cost triaxial anisotropic magneto-resistive sensors. A novel fixed threshold state machine algorithm based on signal variance is proposed. The experimental results have shown that the detection accuracy and average classification accuracy can reach up to 99.05% and 93.66%, respectively. Taghvaeeyan et al. [168] focused on the development of a portable roadside magnetic sensor system for VC. It is shown that the sensor system can count the number of right turns at an intersection, with an accuracy of 95%.

### 2.4. Pneumatic Tubes and other Sensors

Pneumatic tubes are widely used for temporary traffic counts. To collect information regarding vehicle speed and axles, it is necessary to extend two or multiple tubes with an appropriate distance between each other. Pneumatic tubes are easily portable and can be simply placed on the top of road surfaces across travel lanes. The tubes are fixed with pavement nails or other fixtures. These tubes are commercially available for bicycle classification and volume counting. Two studies conducted their research by adapting pneumatic tubes [169,170].

Piezoelectric sensors are made of materials that convert pressure to electrical charges in response to vibrations or mechanical impacts. Piezoelectric sensors are embedded below the pavement surface at each lane, covered with flush epoxy resin for traffic counting and to estimate axle spacing. Furthermore, vehicle speed and inter-axle distance can be determined when two piezoelectric sensors are activated by the same vehicle. These sensors can operate alone or within a WIM system. The generated signals from piezoelectric sensors are collected in a junction box at the roadside. Piezoelectric sensors are sensitive to temperature and surface conditions due to voltage variations. Rajab et al. [19] presented a VC technology by utilizing a single-element piezoelectric sensor placed diagonally on a traffic lane. Diagonally placed piezoelectric strip sensors and machine learning techniques are used to accurately classify vehicles. Testing on several highway sites indicated up to 97% classification accuracy. Santoso et al. [171] proposed a piezoelectric sensor system for measuring traffic flow. A piezoelectric sensor system made of Polyvinylidene Fluoride (PVDF) film, plastered with metal electrodes, for data acquisition and transferring measurement data is introduced for measuring traffic flow. The output shows the number and type of vehicles in the form of a digital code.

Strain gauge sensors are embedded in the structure to measure the strain response of the pavement. The patterns in the dynamic strain response are different for various vehicles; thus, by using pattern recognition and classification methods, the correct group of vehicles can be distinguished. Al-Tarawneh et al. [172] developed a VC system based on novel in-pavement fiber optic Bragg grating (FBG) sensors. Strain change was monitored by the embedded 3-D Glass Fiber-Reinforced Polymer-Packaged Fiber Bragg Grating Sensors (3-D GFRP-FBG) sensors. The VC system was comprised of SVM learning algorithms. The field testing results from real traffic data show that the developed system could accurately estimate VC with 98.5% accuracy.

Seismic sensors are used to capture ground vibrations generated by moving vehicles. Networks of seismic sensors are used to collect data to localize and identify vehicle types. Du et al. [173] applied the Fractal Dimension (FD) to extract the features of the seismic signals for ground targets. The FD is based on a morphological covering (MC) method to extract the features of the seismic signals for ground target classification. The experimental results demonstrated that the proposed methods achieved 90% accuracy for VC. Zhou et al. [174] introduced a feature extracted from ground vehicle-induced seismic signals. This feature was extracted from seismic signals using short-time power spectral density (STPSD) from wheeled and tracked vehicle distinction. It was verified using mixed datasets from field experiments and the SensIT that is a platform for wireless vehicle detection. Table 5 shows the advantages and disadvantages of each VC method.

## 3. Potential Smart Vehicle-Assisted Technologies

Embedded sensors, onboard hardware devices, and intelligent antenna systems mounted on a vehicle for transmitting and receiving signals have provided a unique combination of properties, making smart vehicles as an attractive choice for many high-tech applications. VC can greatly benefit from these technologies. The objective and purpose of this research is to study the capability of different vehicle-assisted techniques to extract the kinematic and physical characteristics of vehicles in real time and in a global manner. This information can be used for a wide variety of applications such as parking management, traffic control, safety, and accident avoidance [149].

Vehicular networks are an emerging technology for intelligent transportation systems to facilitate communication among adjacent vehicles within urban and highway scenarios. VANETs are a class of mobile sensor networks in which vehicles along the road behave as mobile sensor nodes [175]. The application of VANETs aims to make the vehicles equipped with an onboard unit (OBU), to enable them connecting to the global network of vehicles, evoking collaborations with each other and with the nearby wireless infrastructure for data sharing [175]. The synergistic links between the two worlds of VANETs and smart vehicles are highly promising for achieving further on-road safety and benefits for end-users [176]. Internet of Vehicles (IoV) is a typical application of the Internet of Things (IoT) in the field of transportation that is achieved by expanding the capabilities of VANETs.

### 3.1. VANET-Based Methods

VANET is a very promising technology that has recently emerged and has been employed for various applications in transportation and traffic engineering [177]. VANET is a mobile network environment that enables communication between vehicles and roadside units (RSUs) for data sharing [175]. A VANET-based traffic information system consists of vehicles, RSUs and Certification Authorities (CA). The system is generally equipped with an OBU, antenna, GPS, and other sensing devices [178]. An OBU is a small computer mounted on a vehicle to integrate computing, positioning, communication, and human interface modules [179]. An OBU may have other interfaces, such as Universal Serial Bus (USB) and Bluetooth, to link it to computing devices (e.g., laptops, smartphones, and Personal Digital Assistants (PDAs)). RSUs are the infrastructure placed along the roadside to provide V2V connectivity [180]. V2V connectivity enables vehicles to share traffic-related information through short-range wireless communication [181]. CAs are responsible for issuing certificates to vehicles, which can be in the form of electronic licenses and anonymous key pairs [182]. In a VANET system, CAs can be governmental transportation authorities or vehicle manufacturers [182]. The research on VANET has gained intensive interest from both academia and industry over the years. With the use of VANET, large amounts of data can be collected, which are further discussed below.

In a VANET system, the mobility information—e.g., position, traveling lane, speed, and acceleration and deceleration—as well as the physical characteristic parameters of vehicles—e.g., weight, height and length—are used for a wide variety of applications such as parking management, traffic control, safety, and accident avoidance [183]. In the following sections, the extracted information through using VANET is further discussed.

#### 3.1.1. Mobility Parameters of Vehicles

GPS receivers are widely used in VANET’s localization systems to extract mobility information, including position, traveling lane [184], speed, acceleration and deceleration [23,185,186]. Padron et al. [187] introduced a VANET-based collaborative system equipped with a GPS device, a real time clock, and a wireless communication device to broadcast their kinematic parameters, such as current location, speed and direction. Shao et al. [188] proposed cooperative vehicle localization in highway scenarios using the kinematic parameters of all vehicles in a cluster. The proposed method considers acceleration and deceleration as well as the other kinematic parameters to obtain more accurate results. Nayak et al. [189] proposed a position-based high-speed vehicle detection algorithm in VANET. The proposed algorithm determines the driving lane of a vehicle and detects speed violations based on the permitted speed in that lane. The lane changes of a vehicle are also considered using the directional indicators of the vehicle. VANET can deliver the profile of the vehicle and driver in a secure and trusted manner.

GPS receivers can be installed easily in vehicles. However, GPS receivers are not the best solution in these cases, due to their low accuracy range—i.e., up to 20 or 30 m—and limitations when working in indoors or in dense urban areas where there is no direct visibility to satellites. For these reasons, GPS information is likely to be combined with other localization techniques such as dead reckoning [190,191], cellular localization [192,193], and image/video localization [194]. This combination of localization information from different sources can be integrated using data fusion techniques [195,196,197,198]. In VANETs, vehicles periodically send beacons to convey information about their identity, velocity, acceleration, and position. The truthful positioning of nodes is essential for proper application. Boeira et al. [199] designed a fifth-generation wireless scheme for node positioning.

Vehicles in a VANET using GPS signals may face the deterioration or complete loss of GPS signals due to high speed or congestion. Wisitpongphan et al. [200] developed an extended self-correcting localization algorithm to enhance the positioning accuracy and improve the localization estimation of vehicles over VANET. A weight factor is introduced into the function by incorporating the strength of the received signal with the measured distortion of the process. The obtained results show that the new algorithm contributes to a better and more efficient localization.

Vehicle speed varies depending on the lane and road specification. However, on a highway, the speed can be increased by up to 200 Km/H. Moving at a high speed can affect the efficiency of the routing, quickly outdating the position information. Therefore, Alwan et al. [201] proposed an improvement to the position-based routing mechanisms through the real time estimation of vehicle position and possible changes in the frequency of exchanged data based on the extracted high accuracy position. On the other hand, vehicular traffic congestion is an extremely important challenge that can diminish the effectiveness of underlying communication by causing broadcast storm problems [202]. Broadcast storm problems are scenarios in which an excessive number of broadcast packets cause collisions in the link layer. Wisitpongphan et al. [200] quantified the impact of broadcast storms in VANETs in terms of message delays and packet loss rates. Moreover, schemes are proposed to reduce the packet loss rates by up to 70%, while keeping the delays at acceptable levels.

#### 3.1.2. Physical Characteristic Parameters

Several VC schemes have been proposed in the literature for the identification of vehicles’ physical characteristic parameters. Different types of sensors are currently used for vehicle detection and tracking. However, these sensors can be devised for special routes and may only cover a short distance where the accuracy of the collected information is limited [203]. Image-based VC is another technique using omnidirectional camera aerial images, CCTV, or normal cameras. The image quality can be affected due to precipitation, light variation, or the presence of trees and other vehicles blocking the target vehicles. The images can also be of poor quality and the results may not be reliable enough for vehicle identification [139].

Automatic number plate recognition is a real time system that is used to recognize the license numbers of vehicles automatically. Automatic number plate recognition is a method that uses optical character recognition to read vehicles’ license plates. Automatic number plate recognition methods have a significant error rate and a high transaction processing cost [27,94]. Jain et al. [204] provided a new algorithm for recognizing license plates for traffic surveillance. Mathematical morphology and an artificial neural network (ANN) were applied to improve the localization and character segmentation. It was indicated that the algorithm has 97.06%, 95.10%, and 94.12% classification accuracy for license plate localization, segmentation and character recognition, respectively. Du et al. [205] conducted a comprehensive review of the recent advantages in automatic number plate recognition. Reviews by Puranic et al. [206] and Gaikwad and Borole [207] cover other aspects of automatic number plate recognition.

A vehicle identification number (VIN) acts as a unique identifier that displays a car’s unique features, specifications, and manufacturer. With the VIN code, the model, type and brand of a target vehicle can be determined [208]. Mitra and Mondal [203] proposed two schemes for the identification, authentication, and tracking of vehicles using VIN in a VANET. Some modifications are made to include a larger number of vehicle manufacturers. VIN includes 17 characters within three fields: World Manufacturing Identification (WMI), Vehicle Description Section (VDS), and Vehicle Identifier Section (VIS). The WMI field contains three characters, whereas field two contains six characters and the VIS field contains eight characters.

Jalooli et al. [209] proposed an intelligent advisory speed limit for the highway using VANET. The proposed advisory speed limit system provided exclusive advice for speed limits based on the vehicle’s characteristics, including the vehicle type, size, and safety capabilities, as well as traffic and weather conditions. Alhammad et al. [210] proposed the use of VANET to reserve on-street parking spaces. The request message is sent through the vehicle’s OBU with the necessary information. The information is comprised of vehicle type, size, and registration number, along with the driver’s profile. Each vehicle has a unique identification number that serves as the car’s fingerprint.

### 3.2. Internet of Vehicles (IoV)-Based Methods

Recent predictions related to the connection of devices to the Internet indicated that, by 2020, nearly 25 billion “things” will be linked to the Internet, of which vehicles will constitute a significant portion [203]. The Internet of Things (IoT) is a medium to connect the electronic subsystem to the existing Internet infrastructure. The IoT merges every aspect of our daily lives through smart gadgets, which are linked via the Internet. The IoV is a particular aspect of the IoT brought about by the integration of VANETs and the IoT. In VANETs, a vehicle equipped with OBU can join a cluster and exchange useful information with surrounding vehicles [211]. Vehicles in VANETs are mainly considered as nodes to disseminate messages among a group of vehicles inside a region of interest [203]. However, in the IoV paradigm, vehicles are considered as smart devices with a strong capability for computation, storage, and learning, keeping their communication function operating constantly [212]. Pathak et al. [213] discussed the advantages of using the IoV paradigm in an intelligent transportation system and investigated obstacles for the successful implementation of IoT. A novel IoV-based transportation architecture was proposed to manage shipping and handling scenarios. The results of the comparative study showed that the cost of the proposed method is 50% less than conventional methods.

Intelligent transportation systems play an important role in enhancing quality and interactivity and can significantly reduce costs and wastage, as well as improve the traffic management capability in an urban transportation system. Wang et al. [214] established a weighted model for IoV sensing networks using a real-world taxi GPS dataset. Moreover, an IoV-aided local traffic information collection architecture is proposed for optimal traffic information transmission. The simulation results and theoretical analysis show the efficiency and feasibility of our proposed models. Gu et al. [215] discussed an IoV localization algorithm and proposed a method to enhance it by using optimization techniques. The results showed a large enhancement in the precision and reliability of the conventional methods.

## 4. Future Works

This review of the available literature reveals that the number of publications on VC methods did not show a significant improvement over the past decade. While the publication rate is somewhat erratic, the general trend depicted a slight decline in some of the areas. Moreover, it is shown that the available methods are incapable of providing a real time performance for the detection, tracking, and identification of a target vehicle in a global manner. Future work in the field of VC will probably focus on developing collaborative systems that run in the wireless ad hoc networks of fixed or mobile computing devices that rely on vehicular sensors such as cameras, LiDAR, radars, ultrasonic, and other onboard diagnostic sensors.

The currently available methods, except GPS-based ones, failed to produce the global parameters of target vehicles. However, the applied GPS-based VC methods are only capable of providing real time kinematic characteristics of vehicles, while their physical parameters such as shape, axle data and weight are not revealed in GPS-based methods. Moreover, to the best of the authors’ knowledge, no research work on GPS exists in the literature that comprehensively addresses a standard framework completely devoted to dealing with the requirements of VC. Smartphone and GPS apparatuses are the devices used in several studies to extract mobility information. However, these devices are not advised for routine use as reliable tools for determining traffic load. Therefore, future studies should focus on the applicability of vehicular GPS receivers for VC.

This study aims to open up new research fields by transcending the borders to provide a close collaboration between the different engineering disciplines, such as computer, structure, traffic, and automotive technologies, in order to incorporate smart vehicular methods in VC. Among these methods, the authors strongly believe that VANET plays a groundbreaking role.

## 5. Conclusions

The available VC methods reviewed in this paper have valuable features and potential. However, the concept and technical principles of these methods remained largely unchanged and unchallenged compared to vehicles that enjoyed enormous success in design and manufacturing technologies. Nowadays, autonomous and autopilot vehicles are fleeting on the roads and they are expected to offer even greater future applications. Collecting real time traffic information as well as providing global access to sensor data are two crucial requirements for a reliable VC method. Since intrusive and nonintrusive methods cannot provide global quantities of passing vehicles, they are not suitable for the intended purpose. The only choice is a GPS-based method that can provide global data for the mobility parameters of vehicles. This method offers no solution for extracting the physical parameters of the target vehicle.

The possible solutions to deal with the shortcomings of the conventional VC method, in that it is incapable of obtaining global real time vehicular parameters, were studied. The use of vehicular methods provided the basis for the potential classification platform. Finally, VANETs were selected due to their outstanding capabilities in the tracking, detection, and classification of on-road vehicles. The literature on VANETs was reviewed and their advantages and disadvantages for VC were studied. The results show that the mobility parameters of vehicles, including speed, direction, locus, and acceleration/deceleration are accessible by using VANETs. Furthermore, it was demonstrated that the VINs for vehicles within the target area could be accessible for authorities, as they contain information on the brand, model and age of the car.

## Figures and Tables

**Table 1:** A summary of the existing definitions for vehicle classification (VC) phenomenon.

Definition	Reference
“Vehicle classification is the process of separating vehicles according to various predefined classes”.	[29,30]
“Vehicle Classification is to classify all detected vehicles into their specific sub-classes”.	[31]
“Vehicle classification is used to classify vehicles into categories in order to provide information of vehicle’s types that pass the monitoring area”.	[11]
“Vehicle classification is to categorize the detected vehicles into their respective types”.	[32]
“Vehicle classification is one of the many ways to identify a vehicle”.	[33]
“Vehicle classification is an important part of intelligent transportation systems by enabling collection of valuable information for various applications, such as road surveillance and system planning”.	[34]
“Vehicle classification is performed by estimating the size or shape of a passing vehicle”.	[35]
“Vehicle classification is the classification of the vehicle into one of a number of distinct groups”.	[36]

**Table 2:** The five stages of autonomy in smart vehicles.

Level	Autonomy Level	Role of the Human Driver	Example
0	No automation	Completely controlled by driver.	Sensors may provide alarms.
1	Driver assistance	Driver controls the vehicle but some driving assistance features are available.	Adaptive cruise control, parking assistance and lane-keeping assistance.
2	Partial automation	Driver must remain engaged for any intervene on notice. Contact between the driver’s hands and the wheel is necessary.	Adaptive cruise control with lane-changing ability.
3	Conditional automation	Driver is necessary, an autonomous system is available for occasional full control such as emergency braking but the driver must be ready to take control.	Traffic jam pilot.
4	High automation	No driver control is required. This is for specific areas and circumstances such as traffic jams. Driver control is optional.	Autonomous driving in some parts of a city.
5	Full automation	The vehicle can perform all functions under all conditions. The driving wheel is optional.	-

**Table 3:** Summary of the literature reviews on VC.

Reference	Detection Medium							
	Vision	GPS	Sound	Magnetic	Contact	Hybrid	Vibration	Smart Vehicle
Shukla and Saini [83]	✓	✕	✕	✕	✕	✕	✕	✕
Yousaf et al. [84]	✓	✕	✕	✕	✕	✕	✕	✕
Jain et al. [85]	✓	✕	✓	✓	✓	✓	✓	✕
Daigavane et al. [86]	✓	✕	✕	✕	✕	✕	✕	✕
Buch et al. [63]	✓	✕	✕	✕	✕	✕	✕	✕
Abdulrahim and Salam [87]	✓	✕	✕	✕	✕	✕	✕	✕
Chandran and Raman [88]	✓	✕	✕	✕	✕	✕	✕	✕
Hadi et al. [89]	✓	✕	✕	✕	✕	✕	✕	✕
Atiq et al. [90]	✓	✕	✕	✕	✕	✕	✕	✕
Mokha and Kumar [91]	✓	✕	✕	✕	✕	✕	✕	✕
Chandran and Raman [88]	✓	✕	✕	✕	✕	✕	✕	✕
Narhe and Nagmode [92]	✓	✕	✕	✕	✕	✕	✕	✕
Moussa [93]	✓	✕	✕	✕	✕	✕	✕	✕
Bhardwaj and Mahajan [94]	✓	✕	✕	✕	✕	✕	✕	✕
Misman and Awang [95]	✓	✕	✕	✕	✕	✕	✕	✕
Ahmed et al. [96]	✓	✕	✕	✕	✕	✕	✕	✕
Borkar and Malik [97]	✕	✕	✓	✕	✕	✕	✕	✕

**Table 4:** Some of the most common soft-computing methods used for pattern recognition, classification, training or prediction in GPS, video image, aerial images, radar, magnetic sensor-based vehicle classification.

Type of Algorithms	Purpose	Details of the Algorithms
Neural networks	Classification, training, pattern recognition	Recurrent neural networks [16], convolutional neural networks (CNN) [31,103], Recurrent Convolutional Neural Networks (R-CNN), deep neural networks [103], Back-Propagation Neural Network (BPN) [126,130], soft radial basis cellular neural network [131], random neural networks (RNNs) [132], Fast Neural Network (FNN) [133], multi-layer perceptron neural network [134], Radial Basis Function (RBF) neural network [135], backpropagation neural networks [126].
Adaptive Gaussian mixture model (GMM)	Segmentation	Gaussian mixture model [136,137], Recursively updated GMM [105].
Support vector machine (SVM)	-	Multiclass SVM [138], SVM [118,139], linear support vector machine (LSVM) [28,112,140], fuzzy SVM [109], multiclass SVM [12], multi SVM [141], C-SVM [142], kernelled SVM [109], binary SVM [143], Individual SVM (ISVM) [144].

**Table 5:** Pros and cons of VC methods.

Category	Method	Pros and Cons	Count	Speed	Acceleration	Direction	Global Locus	Weight	Axle Configuration	Type and Model	Automatic
Vision-based	Video image detection	Sensitive to environmental conditions; automatic classification, relatively low operational and maintenance costs and high capital cost; non-intrusive, expensive computational burden, privacy concerns.	✓	✓	✓	✓	✕	✕	✕	✓	✓
	Infrared	Low quality of the infrared images; sensitive to environmental conditions; suitable for night vision and precipitation time; generally used for classification of the battlefield vehicles; expensive.	✓	✓	✓	✓	✕	✕	✕	✕	✓
	Radar	Insensitive to inclement weather; somehow inexpensive; non-intrusive; automatic classification; generally not suitable for stop-and-go traffic.	✓	✓	✓	✓	✕	✕	✕	✕	✓
	LiDAR	LiDAR is less expensive to produce and the application is easier than radar. LiDAR does not perform as well as radar in rain and snow.	✓	✓	✓	✓	✕	✕	✕	✕	✓
	Aerial images	Aerial images have high spatial resolution and easier data acquisition. Vehicle detection aerial images is a challenging task due to a large number of objects.	✓	✕	✕	✕	✕	✕	✕	✓	✕
GPS-based methods	Vehicle equipped GPS devices	Need to overcome institutional, privacy and security, and technical challenges; Speeds, accelerations can be obtained by processing GPS data.	✕	✓	✓	✓	✓	✕	✕	✕	✕
	Smartphone or cellular phones	Smartphones are equipped with sensors like accelerometers; gyroscopes, etc. Smartphones are not custom-designed or attached to vehicles’ body thus their relative orientation to the reference vehicle frame may vary all the time.	✕	✓	✓	✓	✓	✕	✕	✕	✕
Sound-based methods	Ultrasonic	Ultrasonic sensors are easy to install, immune to dirt and other contaminants, comparatively less expensive but are weather-sensitive and cannot determine the orientation, type, or brand of the target vehicle.	✓	✕	✕	✕	✕	✕	✕	✕	✓
	Acoustic	Acoustic sensors are low cost, simple and non-intrusive, but at the same time, they require a sophisticated algorithm to extract useful information not. Moreover, they are not suitable for stop-and-go traffic.	✓	✓	✓	✓	✕	✕	✕	✕	✓
Magnetic field	Magnetic sensors	Magnetic sensors are small size, relatively low cost, and less sensitive to capricious weather conditions, noise and Doppler effects. Magnetic sensors are not absolute, so they need to be calibrated.	✓	✓	✓	✓	✕	✕	✕	✕	✓
	Inductive loops	Inductive loops are low-cost solutions but they need a long installation process, and sensor installation is intrusive.	✓	✓	✓	✓	✕	✕	✕	✕	✓
Contact and vibration	Pneumatic	Pneumatic tubes are black, deform easily, and have a low profile. Pneumatic tubes are generally used for temporary traffic counts, and have a modest capability for VC.	✓	✓	✓	✓	✕	✓	✓	✕	✓
	Piezoelectric	Piezoelectric sensors are independent time and speed. Piezoelectric sensors are sensitive to temperature changes.	✓	✓	✓	✓	✕	✕	✓	✕	✓
	Fiber optic	Fiber optic sensors are small, low weight, have a large bandwidth and immune to electromagnetic interfaces. Fiber optic sensors have a limited range of angles that it can sense.									
	Strain gauge	Strain gauges are subject to challenges regarding the adhesion of the sensors and compensation for temperature drift.	✓	✓	✓	✓	✕	✕	✓	✕	✓
	Seismic and vibration	Seismic and vibration sensors provide a good detection range but they need very careful calibration.	✓	✕	✕	✕	✕	✕	✕	✕	✓
Manual	Manual observation	No problems or ambiguities in the manual counts; however, it is time-consuming and labor-intensive.	✓	✕	✕	✓	✕	✕	✓	✓	✕
Multi detection	WIM	WIM systems are safe, efficient, and provide a continuous method for collecting traffic. WIM are expensive and provide low accuracy for estimating weight.	✓	✓	✓	✓	✕	✓	✓	✕	✓
